# *Orthrozanclus elongata* n. sp. and the significance of sclerite-covered taxa for early trochozoan evolution

**DOI:** 10.1038/s41598-017-16304-6

**Published:** 2017-11-24

**Authors:** Fangchen Zhao, Martin R. Smith, Zongjun Yin, Han Zeng, Guoxiang Li, Maoyan Zhu

**Affiliations:** 1 State Key Laboratory of Palaeobiology and Stratigraphy, Nanjing Institute of Geology and Palaeontology, Chinese Academy of Sciences, 39 East Beijing Road, Nanjing, 210008 China; 2 Department of Earth Sciences, Durham University, Durham, DH1 3LE UK; 3 College of Earth Sciences, University of Chinese Academy of Sciences, 19 Yuquan Road, Beijing, 100049 China

## Abstract

*Orthrozanclus* is a shell-bearing, sclerite covered Cambrian organism of uncertain taxonomic affinity, seemingly representing an intermediate between its fellow problematica *Wiwaxia* and *Halkieria*. Attempts to group these slug-like taxa into a single ‘halwaxiid’ clade nevertheless present structural and evolutionary difficulties. Here we report a new species of *Orthrozanclus* from the early Cambrian Chengjiang Lagerstätte. The scleritome arrangement and constitution in this material corroborates the link between *Orthrozanclus* and *Halkieria*, but not with *Wiwaxia* — and calls into question its purported relationship with molluscs. Instead, the tripartite construction of the halkieriid scleritome finds a more compelling parallel in the camenellan tommotiids, relatives of the brachiopods and phoronids. Such a phylogenetic position would indicate the presence of a scleritome in the common ancestor of the three major trochozoan lineages, Mollusca, Annelida and Brachiozoa. On this view, the absence of fossil Ediacaran sclerites is evidence against any ‘Precambrian prelude’ to the explosive diversification of these phyla in the Cambrian, c. 540–530 million years ago.

## Introduction

The Cambrian fossil record is renowned for the morphologically puzzling organisms that it preserves. Such taxa often represent long-extinct combinations of characters, offering a unique perspective on the early origin of modern body plans – presuming, of course, that relationships with modern groups can be established^1^. The reconstructed origins of the molluscan lineage, for example, have been overhauled in order to accommodate two emblematic Cambrian taxa, *Halkieria* and *Wiwaxia* ^2–9^. These two genera bear superficially similar sclerites, which occur the world over as carbonaceous and mineralized microfossils^10–13^; the grouping Sachitida was erected to reflect this perceived commonality^14^. The case for phylogenetic proximity was strengthened by the discovery of articulated specimens in the Burgess Shale and Sirius Passet *Lagerstätten*, which showed that the sclerites of both taxa were dorsal and imbricating^2,15,16^. This arguably overlooks some notable differences between the two genera – *Halkieria* has dorsal valves, *Wiwaxia* bears elongate spines, and the sclerites of the two groups are far from identical – but suggestions that these differences might denote a degree of phylogenetic separation^17,18^ were soon countered by the description of the Burgess Shale animal *Orthrozanclus reburrus*, which incorporates a single *Halkieria*-like valve within a spiny non-mineralized scleritome^19^. The ‘halwaxiid’ clade, incorporating *Wiwaxia*, *Orthrozanclus*, *Halkieria* and other sachitids, was erected on the basis that the scleritomes of these taxa were consequently homologous. A new species of *Orthrozanclus* from the Chengjiang lagerstätten, however, prompts a re-evaluation of the basis for a halwaxiid grouping, and calls into question the position of *Halkieria* and *Orthrozanclus* in molluscan evolution.

## Results

### Systematic Palaeontology

Superphylum Lophotrochozoa

Family Halkieriidae Poulsen 1967^20^.

#### Remarks

*Orthrozanclus* falls within the emended diagnosis of Halkieriidae provided by Conway Morris and Peel 1995^16^, negating the need for a separate family Orthrozanclidae^19^.

*Orthrozanclus* Conway Morris and Caron 2007^19^

**Figure 1:**
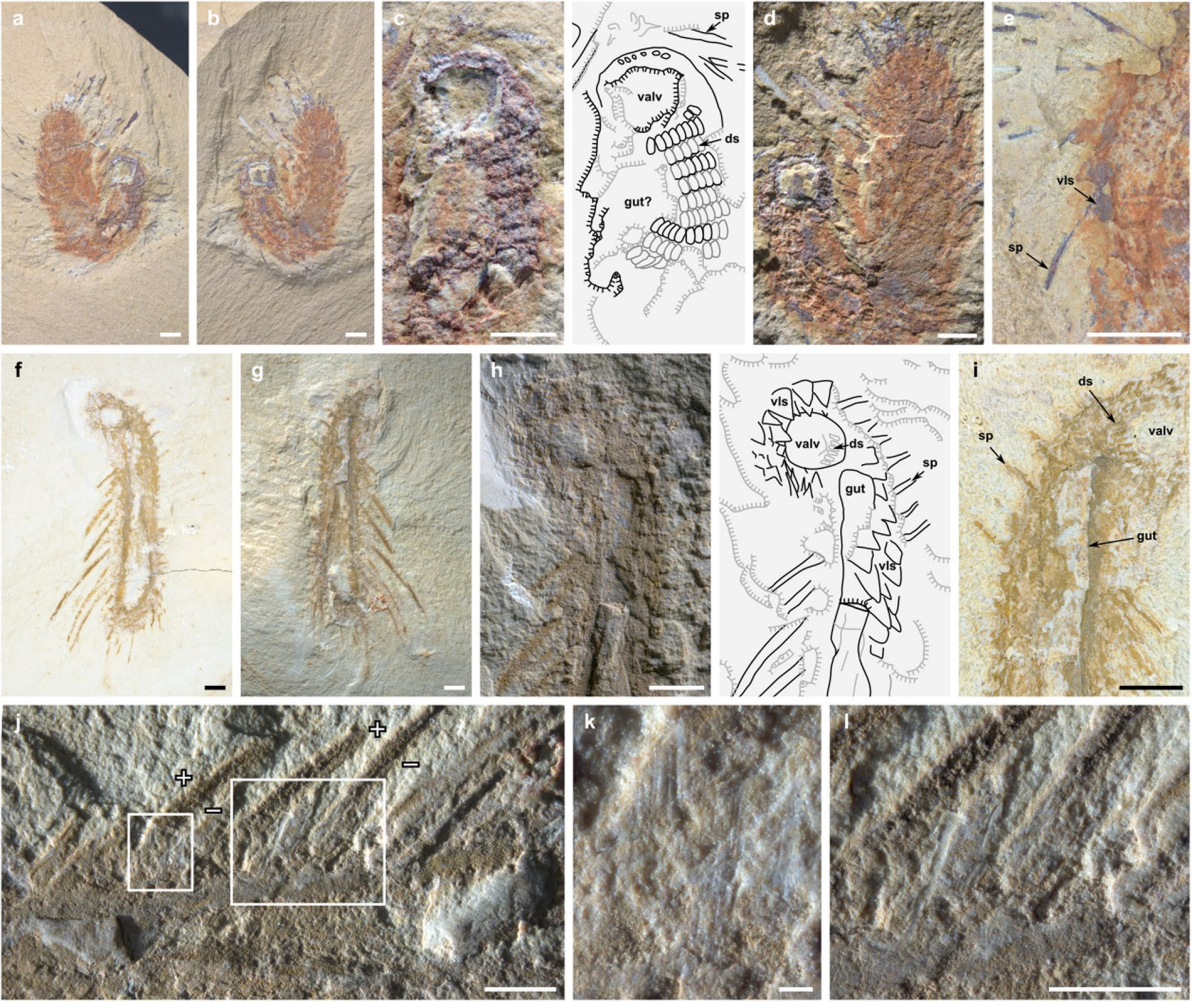
*Orthrozanclus elongata* n. sp. (**a**–**e**) NIGPAS164893, paratype. (**a**,**b**) part and counterpart of entire specimen. (**c**) part, anterior region, dorsal sclerites exhibit relief. (**d**) counterpart, showing ‘fanning’ of spines at posterior. (**e**) counterpart, showing arrangement of spines and ventrolateral sclerites. (**f–l**) NIGPAS164892, holotype. (**f**,**g**) part and counterpart of entire specimen. (**h**) part, anterior region, dark field illumination emphasizes relief of ventrolateral sclerites. (**i**) counterpart, anterior region, bright field illumination emphasizes sclerite margins. (**j**) counterpart, left lateral region showing inclination of spines relative to the bedding plane – the anterior edge (+) is raised above the posterior edge (−) – and ribs on ventrolateral sclerites (**k**) and dorsolateral spines (**l**). Abbreviations: ds, dorsal sclerites; sp, spines; valv, valve; vls, ventrolateral sclerites. Bars = 1 mm except k, 100 µm.

**Figure 2:**
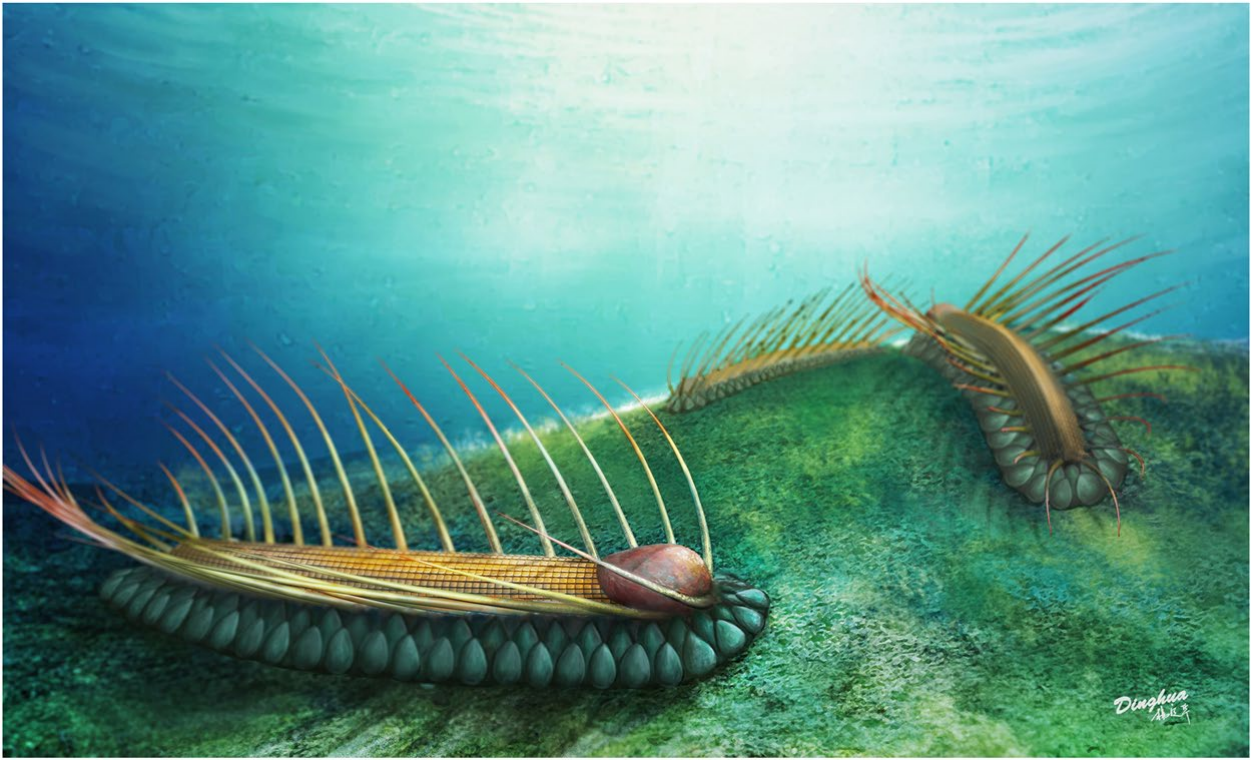
Reconstruction of *Orthrozanclus elongata* n. sp. in life.

*Orthrozanclus elongata* Zhao et Smith n. sp. Figs 1 and 2.

#### Type material

NIGPAS 164892 (Fig. 1f–l), holotype; 164893 (Fig. 1a–e), paratype, each comprising part and counterpart and preserved in the characteristic Chengjiang fashion^21^ as weathered aluminosilicate films associated with superficial iron oxides.

#### Provenance

Maotianshan Shale, Yu’anshan Formation, *Eoredlichia-Wutingaspis* Zone, Cambrian Series 2, Stage 3. The holotype was collected from Jiucun, near Chengjiang (24°41’33” N, 102°59’26” E); the paratype from Yuanbaocun, Chenggong, Kunming (24°49’24” N, 102°49’14” E), Yunnan, southwest China.

#### Diagnosis

Species of *Orthrozanclus* with elongate (c. 1:7) aspect ratio. Dorsal sclerites mineralized, oblong in aspect, occurring in regular rows. Dorsolateral spinose sclerites flat, ribbed and blade-like, without central cavity.

#### Description

The two specimens of *Orthrozanclus elongata* n. sp. (Fig. 1) are 20 mm long and a uniform 3 mm in width. Their dorsal scleritome bears an anterior valve and three zones of sclerites: a medial zone covers the flattened dorsal surface of the organism, and inner and outer peripheral zones surround its flanks. Its rectangular outline, rounded anterior and posterior ends and overall architecture resemble that of *O. reburrus*.

The medial sclerite zone comprises transverse chevron-like rows, each containing fourteen sclerites, seven on each side (Figs 1c and 2). These sclerites measure 220 × 90 µm, are oblong to teardrop shaped, and lie flat to the body. Their pronounced three-dimensionality distinguishes these sclerites from those in other zones, and – in view of the well-defined margins of the individual sclerites – indicates an originally mineralized composition. Neither phosphatization of labile tissue^22^ nor secondary infilling of original cavities (as observed in *Wiwaxia* and *O. reburrus* ^8,19^) are consistent with the observed preservation. The enhanced relief of the dorsal elements relative to the dorsolateral and ventral sclerites presumably reflects original three-dimensional structure.

The spinose dorsolateral sclerites reach 6 mm in length, and form a c. 45° angle to the body, with their tips directed posteriad (Figs 1 and 2). They are regularly spaced (Fig. 1h–j) in a single series that encircles the body, surrounding the anterior margin of the valve and the posterior of the dorsal area (Fig. 1d). The spines bear ribs, but are otherwise flat in cross-section; in contrast to *O. reburrus*, there is no evidence of a central cavity (Fig. 1j–l). Their flat surfaces lie at an angle of 20–45° to the bedding surfaces – indicating a high original angle (Fig. 1j). Apparent differences in width between spines can be attributed to differential angles of burial relative to the bedding surface. The proximal configuration of the spines (Fig. 1l) has a putative similarity to the auricle of certain *Halkieria* sclerites^10^.

Dagger-shaped (cultrate) sclerites occupy the lateral surfaces of the organism, extending to partly enclose the ventral surface (Fig. 1h). The best-preserved sclerites bear a bilaterally symmetrical series of ribs (Fig. 1k). These sclerites (but not the spines or dorsal sclerites) encircle the valve to enclose the anterior margin of the organism (Fig. 1h); the tips of the sclerites were originally directed dorsally, rather than radially as depicted for *O. reburrus*.

The valve is denoted by a region of pronounced relief, presumably reflecting a robustly mineralized original constitution (Fig. 1c–d,h). The shape of the valve suggests a posterior umbo: though the opposite has been interpreted in *O. reburrus*, the umbo is difficult to locate with certainty in either taxon. The posterior and anterior margins of the valve are overlapped by sclerites of the medial and outer peripheral zones respectively (Fig. 1h).

**Figure 3:**
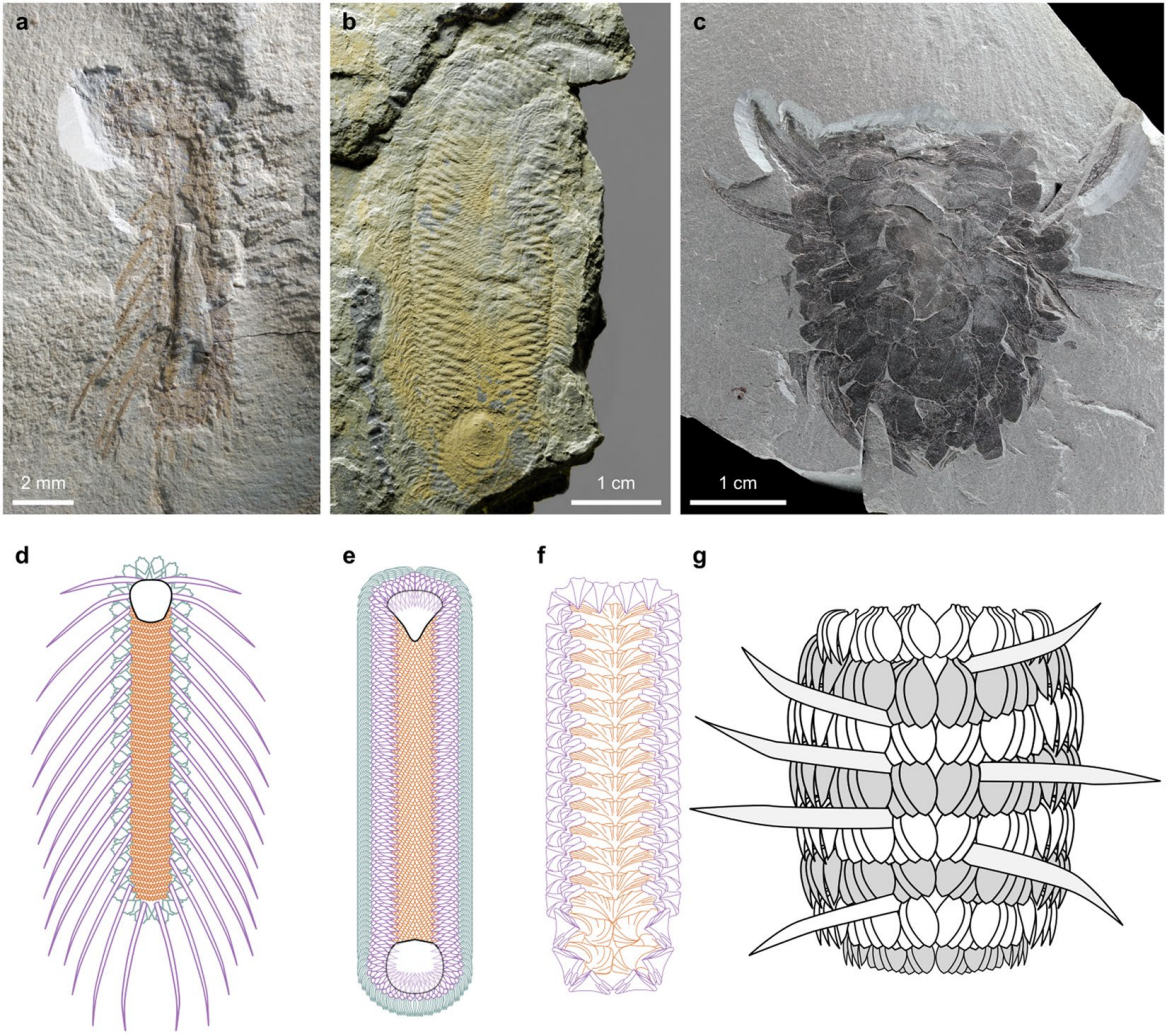
Scleritome arrangement in *Orthrozanclus elongata* n. sp. (**a**, NIGPAS164892), *Halkieria evangelista* (**b**, Sedgwick Museum of Earth Sciences  X24914.2) and *Wiwaxia corrugata* (c, Royal Ontario Museum 61510). The *Orthrozanclus* (**d**) and *Halkieria* (**e**) scleritomes are arranged in three concentric zones: a medial zone of oblique transverse rows (vermillion); a dorsolateral ‘inner peripheral’ zone (purple), containing long spines in *Orthrozanclus* and cultrate sclerites in *Halkieria*; and a ventrolateral ‘outer peripheral’ zone, containing cultrate (*Orthrozanclus*) or siculate (*Halkieria*) sclerites. *Dailyatia bacata* (**f**) is reconstructed as having a medial region containing A and B sclerites and a single peripheral zone of C sclerites. The approximately 4:1 ratio of C1:A sclerites and 4:2 ratio of C2:B sclerites^35^ is taken to indicate that C sclerites occur at twice the frequency of elements in the medial zone. The *Wiwaxia* scleritome (**g**) comprises eight transverse rows (shaded) intersected by two rows of intermittently spaced spines.

A three-dimensionally preserved structure, presumably representing the digestive tract, follows the main body axis (Figs. 1d,h and 3a). As with the presumed gut of *O. reburrus*, this begins slightly posterior to the shell; the gap between the gut and the shell marks a 90° bend in the axis of NIGPAS 164892, reminiscent of an equivalent bend in many *Halkieria* fossils (see ref.^16^ and Fig. 3b).

## Discussion

The new material strengthens the case for a close relationship between *Orthrozanclus* and *Halkieria* (Fig. 3). Mineralized dorsal sclerites, occurring in oblique transverse rows behind an anterior shell, are now evident in both taxa (Fig. 3a–b,d–e) – even if *Orthrozanclus* has no counterpart to the posterior shell of *Halkieria*. And each taxon exhibits two peripheral sclerite zones: the inner, dorsolateral zone contains long spines in *Orthrozanclus* and short cultrate sclerites in *Halkieria*; the outer, ventrolateral zone bears regularly spaced cultrate sclerites in *Orthrozanclus* and siculate sclerites in *Halkieria* (Fig. 3a–b,d–e). Homology of the zones is thus recognized based on their position, rather than the shape or constitution of the sclerites that they contain.

An equivalent sclerite arrangement was once envisaged in *Wiwaxia* ^15,23^, but recent studies^8,9^ have shown that the *Wiwaxia* scleritome conforms to a metameric architecture, comprising 8–9 transverse rows (Fig. 3c,g). Even though the most lateral sclerites are morphologically distinct in certain *Wiwaxia* species, they belong to the same transverse rows as the medial sclerites, rather than forming a distinct peripheral zone that surrounds the entire circumference of the organism^9,24^ (Fig. 3c). The two dorsal rows of spines in *Wiwaxia* are highly variable in their number, size, spacing, and orientation, both within and between species^8,15,25^, so do not form a distinct region of the scleritome architecture. As such, the peripheral sclerite zones in *Orthrozanclus* (Fig. 3a) and *Halkieria* (Fig. 3b) have no counterpart in *Wiwaxia*, and it is not clear that the two scleritome layouts are equivalent in any meaningful way – undermining the case for a ‘halwaxiid’ clade.

### Are halkieriids molluscs?

At a broader taxonomic level, perceived similarities in scleritome construction are said to indicate a close relationship between halkieriids and aculiferan molluscs^4,6,19,26–28^. This position has most recently been propounded based on the Ordovician aculiferan *Calvapilosa*, which has been interpreted as a close relative of halkieriids^28^. The evidence that *Calvapilosa* is an aculiferan is strong; the evidence that it is a halkieriid warrants more careful consideration.

Sclerites – a likely inheritance from the ancestral lophotrochozoan^17,18,29,30^ – have been assembled into scleritomes on multiple occasions: the scleritomes of the scaly-footed gastropods^31^ and chrysopetalid annelids^32,33^, for example, represent independent innovations that are demonstrably unique to the respective clades^31^. Indeed, multiple groups incorporate both shell-like valves and mineralized plates into dorsal imbricating skeletons – witness machaeridians, *Pelagiella* and certain tommotiids, who have affinities with annelids, gastropods and brachiopods, respectively^34–37^.

It is therefore significant that the *Calvapilosa* scleritome prominently lacks the differentiated sclerite morphologies and peripheral morphological zones that characterize halkieriids. Halkieriid sclerites exhibit a broad range of morphologies, but none resemble the slender, spinose sclerites of *Calvapilosa* ^28^. The central cavity present in both halkieriid and *Calvapilosa* sclerites has little taxonomic value (discussed in ref.^8^). The shell of *Calvapilosa* is a markedly different shape to that of *Halkieria*, and bears depressions (interpreted as aesthete canals) that have no counterpart in halkieriid shells.

In the absence of any demonstrably equivalent constructional features or an unambiguously close genetic relationship, it is difficult to defend the homology of the halkieriid scleritome with that of *Calvapilosa*.

One thing that *Calvapilosa* (and *Wiwaxia* ^7^) does establish is that where a radula is present, it preserves readily in Burgess Shale-type conditions. But importantly, this robust and distinctive multi-row mouthpart is prominently absent in both *Orthrozanclus* and *Halkieria*. (A potentially radula-like structure evident in a single specimen of *Halkieria* ^16^ corresponds in angle and dimensions with diagonal displacements of sclerites elsewhere in the scleritome, and is not associated with any diagnostically radular characteristics, such as teeth^38^; its identification as a radula must be considered unproven.) As a radula was present in the ancestral mollusc^39^, and perhaps deeper in the trochozoan lineage^9^, its absence in halkieriids is difficult to reconcile with a molluscan affinity.

### Could halkieriids be tommotiids?

**Figure 4:**
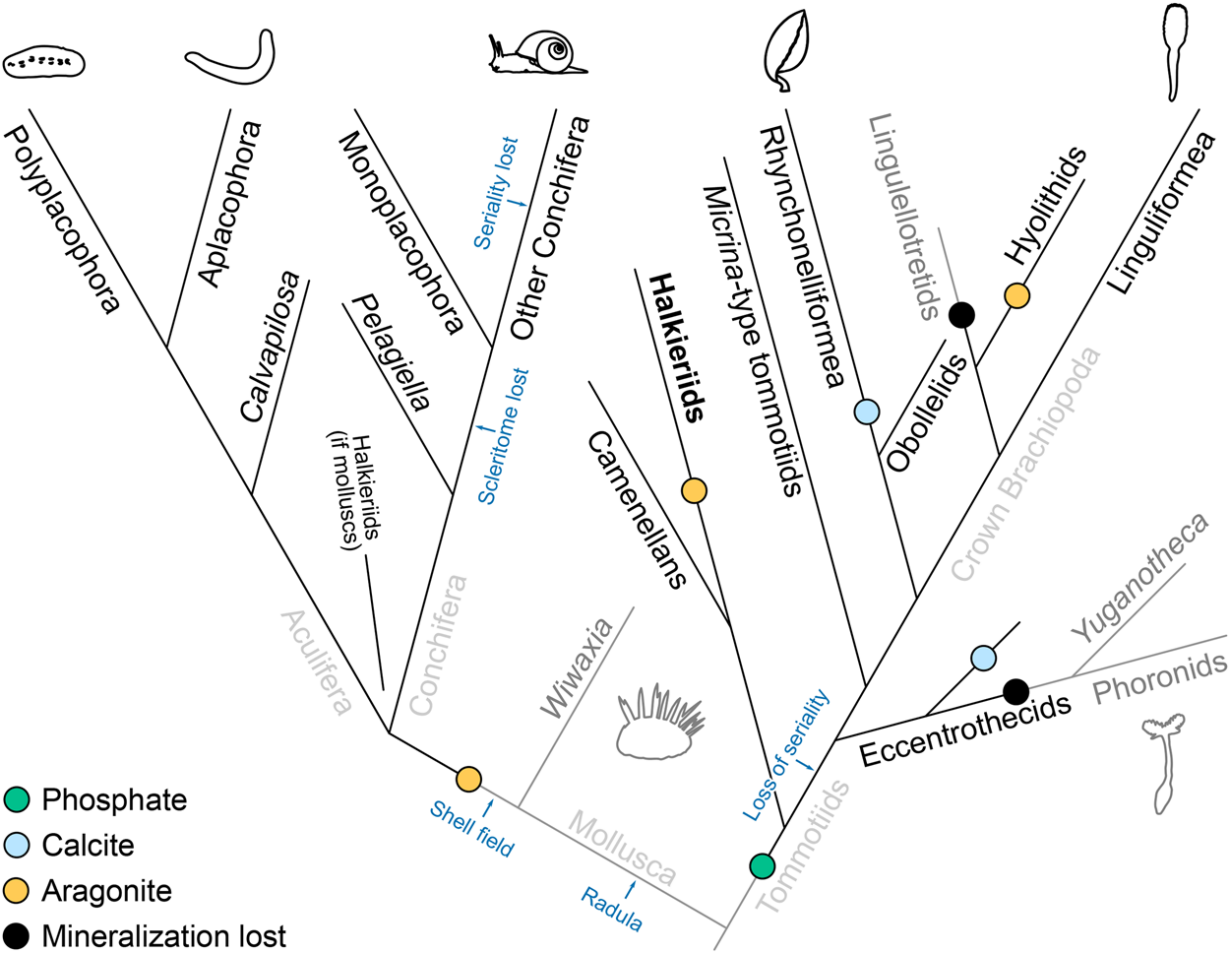
Possible position of halkieriids within tommotiids. The common ancestor of Trochozoa is reconstructed as a non-mineralizing scleritomous organism with serially repeated elements. The presence of biomineralized elements is denoted by line colour, with changes in biomineral marked by circles.

One set of organisms whose scleritomes exhibit an intriguing similarity with those of halkieriids are the camenellan tommotiids, a group that is implicated in the earliest ancestry of brachiopods^40–43^. The scleritome of the kennardiid camenellan *Dailyatia* ^35^ has been reconstructed as comprising median and peripheral fields (Fig. 3f). The medial region bears a series of transverse ‘rows’ of one or two sclerites (A and B sclerites); the peripheral field bears dorsally-directed sclerites with a distinct morphology (C sclerites). As no fully articulated camenellan scleritomes have yet been found, this comparison does of course warrant a degree of caution, particularly in view of the tube-like configuration of other tommotiid scleritomes^37,44–46^ – but the general arrangement reconstructed from sclerite asymmetry, fused arrays of sclerites, morphological proportions and relative sclerite frequency is fundamentally compatible with a halkieriid-like construction. Taking this further, sclerites in the peripheral zones of camenellan scleritomes occur in dextral and sinistral forms^35,47^, as do the sclerites of *Halkieria* ^10^ and – in view of the symmetrical scleritome arrangement revealed by *O. elongata* n. sp. – those of *Orthrozanclus*. Camenellan sclerites show continuous variation within a particular morphological category^47^ – as do spines in the dorsolateral zone of the *Orthrozanclus* scleritome. Certain camenellan sclerites^48^ exhibit a tuberculate ornament and apical tip that correspond closely to the sclerites of, for example, *Halkieria mira* (see Figs 4, 6 in ref.^49^). More speculatively, the camerate construction of certain halkieriid sclerites^6,10^ might find a parallel in the internal chambers of *Kelanella* sclerites or *Micrina* valves^47,50^.

Looking more widely, the paired muscle scars and shelly internal projections evident in Morph A valves of *Oikozetetes* ^51,52^, some of the best documented halkieriid shells, have possible parallels in the equivalent paired muscle scars and internal processes present in the mitral sclerite of the tommotiids *Micrina* ^44^ and *Dailyatia* ^35^ and the operculum of hyolithids^53^ (potential relatives of tommotiids^54^).

In view of these similarities, we therefore propose that halkieriids and camenellans may be closely related (Fig. 4). If camenellans are derived from an ancestrally tube-dwelling tommotiid^55^, then a vagrant, slug-like habit would represent an apomorphy of a halkieriid + camenellan clade; alternatively, the halkieriid condition may be ancestral for the tommotiid + brachiopod lineage^16,40^, with the bivalved condition perhaps arising through paedomorphic retention of an ancestral state^41^.

One obvious objection to this taxonomic hypothesis is that camenellan elements are composed of calcium phosphate, whereas halkieriids secreted calcium carbonate, probably in the form of aragonite^56^. This said, tommotiids and early brachiopods deploy a wide variety of biominerals (Fig. 4): examples exist of non-mineralized, agglutinated, aragonitic, calcitic, phosphatic, and mixed calcite-phosphate shells^54,57,58^.

Switching from one biomineral to another is generally the exception rather than the rule^59,60^, but members of the brachiopod lineage have nevertheless changed their primary biomineral from phosphate to calcite^61,62^, from calcite to aragonite^63^, and from phosphate to a non-mineralized configuration^64^; indeed, some living brachiopods switch from using silica to calcite as they grow^65^.

On a broader view, biomineralization has evolved multiple times within Metazoa^66^, seemingly coming and going in Ediacaran lineages according to prevailing environmental conditions^67^. If this situation persisted into the early Cambrian, it is possible to envision a predominantly non-mineralised brachiopod stem lineage that obtained biomineralization on multiple occasions, each time reflecting the prevailing seawater chemistry. The aragonite mineralogy of halkieriids and hyoliths arose in the aragonite seas of the Fortunian; the calcitic and phosphatic mineralogies of tommotiids and crown-group brachiopods arose in the calcite seas of the Tommotian^59^. Linguliforms and tommotiid-like specimens from Burgess Shale-type deposits^64,68,69^ attest to the persistence of non-mineralized skeletons across the brachiopod total group into the mid-Cambrian. In any case, whether modification or multiple innovations account for the diversity of biomineral use in brachiopods and tommotiids, the carbonate elements of halkieriids clearly fit within this gamut.

## Conclusion

Because halkieriid-like sclerites occur so early in the Cambrian period^70,71^, their affinity has profound implications for the timing of early trochozoan evolution. Removing halkieriids from Mollusca would shift the origin of this phylum significantly later: notwithstanding hyoliths (now interpreted as brachiozoans, i.e. brachiopods or phoronids^54^) and helcionellids (which lack any compelling molluscan apomorphies), there are no strong candidates for crown group molluscs until the Tommotian, and no unequivocal cases until the Late Cambrian^1^.

If, on the other hand, brachiozoans evolved from a halkieriid-like ancestor, then multi-element scleritomes characterise the earliest brachiozoans as well as molluscs and annelids^9^ (Fig. 4). The absence of such sclerites among Ediacaran and earliest Cambrian fossil assemblages^55^ either requires special taphonomic pleading or genuinely denotes that Trochozoans had not yet originated. The subsequent appearance of a rich diversity of exoskeletal elements in the early Cambrian fossil record^12,72^ points to a very rapid origin and divergence of the key lophotrochozoan phyla in the first few million years of the Cambrian period – representing a truly ‘explosive’ evolutionary radiation.

## Methods

The paratype was prepared with a fine blade. Photographs were taken using a Zeiss Stereo Discovery V16 microscope system and processed using TuFuse and the GNU image manipulation program.

### Data availability

Specimens are accessioned at the Nanjing Institute of Geology and Palaeontology, Chinese Academy of Sciences (NIGPAS); high resolution images are available at the FigShare repository^73^.

## References

[1] Budd GE, Jensen S (2000). A critical reappraisal of the fossil record of the bilaterian phyla. Biol. Rev..

[2] Conway Morris S, Peel JS (1990). Articulated halkieriids from the Lower Cambrian of north Greenland. Nature.

[3] Bengtson S (1992). The cap-shaped Cambrian fossil *Maikhanella* and the relationship between coeloscleritophorans and molluscs. Lethaia.

[4] Vinther J, Nielsen C (2005). The Early Cambrian *Halkieria* is a mollusc. Zool. Scr..

[5] Caron J-B, Scheltema AH, Schander C, Rudkin D (2006). A soft-bodied mollusc with radula from the Middle Cambrian Burgess Shale. Nature.

[6] Vinther J (2009). The canal system in sclerites of Lower Cambrian *Sinosachites* (Halkieriidae: Sachitida): significance for the molluscan affinities of the sachitids. Palaeontology.

[7] Smith MR (2012). Mouthparts of the Burgess Shale fossils *Odontogriphus* and *Wiwaxia*: implications for the ancestral molluscan radula. Proc. R. Soc. B.

[8] Smith MR (2014). Ontogeny, morphology and taxonomy of the soft-bodied Cambrian ‘mollusc’. Wiwaxia. Palaeontology.

[9] Zhang Z-F, Smith MR, Shu D-G (2015). New reconstruction of the *Wiwaxia* scleritome, with data from Chengjiang juveniles. Sci. Rep..

[10] Bengtson S, Conway Morris S (1984). A comparative study of Lower Cambrian *Halkieria* and Middle Cambrian *Wiwaxia*. Lethaia.

[11] Porter SM (2008). Skeletal microstructure indicates chancelloriids and halkieriids are closely related. Palaeontology.

[12] Kouchinsky AV (2012). Chronology of early Cambrian biomineralization. Geol. Mag..

[13] Slater BJ, Harvey THP, Guilbaud R, Butterfield NJ (2017). A cryptic record of Burgess Shale-type diversity from the early Cambrian of Baltica. Palaeontology.

[14] Hao T-G (1981). Lower Cambrian (Meishucunian) sachitids and their stratigraphic significance [in Chinese]. J. Chengdu Coll. Geol..

[15] Conway Morris S (1985). The Middle Cambrian metazoan *Wiwaxia corrugata* (Matthew) from the Burgess Shale and *Ogygopsis* Shale, British Columbia, Canada. Phil. Trans. R. Soc. Lond. B.

[16] Conway Morris S, Peel JS (1995). Articulated halkieriids from the Lower Cambrian of North Greenland and their role in early protostome evolution. Phil. Trans. R. Soc. B.

[17] Butterfield NJ (1990). A reassessment of the enigmatic Burgess Shale fossil *Wiwaxia corrugata* (Matthew) and its relationship to the polychaete *Canadia spinosa* Walcott. Paleobiology.

[18] Butterfield NJ (2006). Hooking some stem-group ‘worms’: fossil lophotrochozoans in the Burgess Shale. BioEssays.

[19] Conway Morris S, Caron J-B (2007). Halwaxiids and the early evolution of the lophotrochozoans. Science.

[20] Poulsen C (1967). Fossils from the Lower Cambrian of Bornholm. Danske Vidensk. Selsk. Mat. Meddelelser.

[21] Zhu M-Y, Babcock LE, Steiner M (2005). Fossilization modes in the Chengjiang Lagerstätte (Cambrian of China): testing the roles of organic preservation and diagenetic alteration in exceptional preservation. Palaeogeogr. Palaeoclimatol. Palaeoecol..

[22] Butterfield NJ (2002). *Leanchoilia* guts and the interpretation of three-dimensional structures in Burgess Shale-type fossils. Paleobiology.

[23] Eibye-Jacobsen D (2004). A reevaluation of *Wiwaxia* and the polychaetes of the Burgess Shale. Lethaia.

[24] Yang J, Smith MR, Lan T, Hou J-B, Zhang X-G (2014). Articulated *Wiwaxia* from the Cambrian Stage 3 Xiaoshiba Lagerstätte. Sci. Rep..

[25] Conway Morris S, Selden PA, Gunther G, Jamison PM, Robison RA (2015). New records of Burgess Shale-type taxa from the middle Cambrian of Utah. J. Paleontol..

[26] Sigwart JD, Sutton MD (2007). Deep molluscan phylogeny: synthesis of palaeontological and neontological data. Proc. R. Soc. B.

[27] Sutton MD, Briggs DEG, Siveter DJ, Siveter DJ, Sigwart JD (2012). A Silurian armoured aplacophoran and implications for molluscan phylogeny. Nature.

[28] Vinther J, Parry L, Briggs DEG, Van Roy P (2017). Ancestral morphology of crown-group molluscs revealed by a new Ordovician stem aculiferan. Nature.

[29] Schiemann SM (2016). Clustered brachiopod Hox genes are not expressed collinearly and are associated with lophotrochozoan novelties. bioRχiv.

[30] Peterson KJ, Eernisse DJ (2001). Animal phylogeny and the ancestry of bilaterians: inferences from morphology and 18S rDNA gene sequences. Evol. Dev..

[31] Chen C, Copley JT, Linse K, Rogers AD, Sigwart J (2015). How the mollusc got its scales: convergent evolution of the molluscan scleritome. Biol. J. Linn. Soc..

[32] Watson Russell C (2000). Description of a new species of *Arichlidon* (Chrysopetalidae: Polychaeta) from the West Atlantic and comparison with the East Atlantic species *Arichlidon reyssi*. Bull. Mar. Sci..

[33] Watson Russell C (1997). Patterns of growth and setal development in the deep-sea worm, *Strepternos didymopyton* (Polychaeta: Chrysopetalidae). Bull. Mar. Sci..

[34] Vinther J, Van Roy P, Briggs DEG (2008). Machaeridians are Palaeozoic armoured annelids. Nature.

[35] Skovsted CB, Betts MJ, Topper TP, Brock GA (2015). The early Cambrian tommotiid genus *Dailyatia* from South Australia. Mem. Assoc. Australas. Palaeontol..

[36] Thomas, R. D. K., Vinther, J. & Matt, K. Structure and evolutionary implications of finely preserved chaetae associated with *Pelagiella*, a stem-group gastropod from the Kinzers Formation (Early Cambrian) at Lancaster, Pennsylvania. In *International Palaeontological Congress 3, London, U.K. Programme & Abstracts* 375 (2010).

[37] Larsson CM (2014). *Paterimitra pyramidalis* from South Australia: scleritome, shell structure and evolution of a lower Cambrian stem group brachiopod. Palaeontology.

[38] Smith MR (2017). Putative ‘radula’ in *Halkieria evangelista*. FigShare.

[39] Scheltema AH (2014). The original molluscan radula and progenesis in Aplacophora revisited. J. Nat. Hist..

[40] Holmer LE, Skovsted CB, Williams A (2002). A stem group brachiopod from the lower Cambrian: support for a *Micrina* (halkieriid) ancestry. Palaeontology.

[41] Holmer LE, Skovsted CB, Larsson C, Brock GA, Zhang Z (2011). First record of a bivalved larval shell in Early Cambrian tommotiids and its phylogenetic significance. Palaeontology.

[42] Balthasar U, Skovsted CB, Holmer LE, Brock GA (2009). Homologous skeletal secretion in tommotiids and brachiopods. Geology.

[43] Harper DAT, Popov LE, Holmer LE (2017). Brachiopods: origin and early history. Palaeontology.

[44] Holmer LE, Skovsted CB, Brock GA, Valentine JL, Paterson JR (2008). The Early Cambrian tommotiid *Micrina*, a sessile bivalved stem group brachiopod. Biol. Lett..

[45] Skovsted CB, Clausen S, Álvaro JJ, Ponlevé D (2014). Tommotiids from the early Cambrian (Series 2, Stage 3) of Morocco and the evolution of the tannuolinid scleritome and setigerous shell structures in stem group brachiopods. Palaeontology.

[46] Skovsted CB, Brock GA, Topper TP, Paterson JR, Holmer LE (2011). Scleritome construction, biofacies, biostratigraphy and systematics of the tommotiid *Eccentrotheca helenia* sp. nov. from the Early Cambrian of South Australia. Palaeontology.

[47] Devaere L (2014). The tommotiid *Kelanella* and associated fauna from the early Cambrian of southern Montagne Noire (France): implications for camenellan phylogeny. Palaeontology.

[48] Devaere L, Skovsted CB (2016). New early Cambrian sclerites of *Lapworthella schodakensis* from NE Greenland: advancements in knowledge of lapworthellid taxonomy, sclerite growth and scleritome organization. Geol. Mag..

[49] Conway Morris S, Chapman AJ (1997). Lower Cambrian halkieriids and other coeloscleritophorans from Aksu-Wushi, Xinjiang, China. J. Paleontol..

[50] Li G-X, Xiao S-H (2004). *Tannuolina* and *Micrina* (Tannuolinidae) from the Lower Cambrian of eastern Yunnan, South China, and their scleritome reconstruction. J. Paleontol..

[51] Paterson JR, Brock GA, Skovsted CB (2009). *Oikozetetes* from the early Cambrian of South Australia: implications for halkieriid affinities and functional morphology. Lethaia.

[52] Jacquet SM, Brock GA, Paterson JR (2014). New data on *Oikozetetes* (Mollusca, Halkieriidae) from the lower Cambrian of South Australia. J. Paleontol..

[53] Martí Mus M, Bergström J (2005). The morphology of hyolithids and its functional implications. Palaeontology.

[54] Moysiuk J, Smith MR, Caron J-B (2017). Hyoliths are Palaeozoic lophophorates. Nature.

[55] Budd GE, Jackson ISC (2016). Ecological innovations in the Cambrian and the origins of the crown group phyla. Phil. Trans. R. Soc. B.

[56] Porter SM (2004). Halkieriids in Middle Cambrian phosphatic limestones from Australia. J. Paleontol..

[57] Balthasar U (2007). An Early Cambrian organophosphatic brachiopod with calcitic granules. Palaeontology.

[58] Zhang Z-F (2014). An early Cambrian agglutinated tubular lophophorate with brachiopod characters. Sci. Rep..

[59] Porter SM (2010). Calcite and aragonite seas and the *de novo* acquisition of carbonate skeletons. Geobiology.

[60] Zhuravlev AY, Wood RA (2008). Eve of biomineralization: Controls on skeletal mineralogy. Geology.

[61] Balthasar U (2008). *Mummpikia* gen. nov. and the origin of calcitic-shelled brachiopods. Palaeontology.

[62] Skovsted CB (2016). A silicified tommotiid from the lower Cambrian of Greenland. Bull. Geosci..

[63] Balthasar U (2011). Relic aragonite from Ordovician-Silurian brachiopods: Implications for the evolution of calcification. Geology.

[64] Holmer LE, Caron J-B (2006). A spinose stem group brachiopod with pedicle from the Middle Cambrian Burgess Shale. Acta Zool..

[65] Williams A, Lüter C, Cusack M (2001). The nature of siliceous mosaics forming the first shell of the brachiopod *Discinisca*. J. Struct. Biol..

[66] Knoll, A. H. In *Reviews in Mineralogy and* Geochemistry (eds Dove, P. M., DeYoreo, J. J. & Weiner, S.) **54**, 329–356 (Mineralogical Society of America, 2003).

[67] Wood R, Ivantsov AY, Zhuravlev AY (2017). First macrobiota biomineralization was environmentally triggered. Proc. R. Soc. B.

[68] Balthasar U, Butterfield NJ (2009). Early Cambrian ‘soft-shelled’ brachiopods as possible stem-group phoronids. Acta Pal. Pol..

[69] Zhang Z-F (2013). A sclerite-bearing stem group entoproct from the early Cambrian and its implications. Sci. Rep..

[70] Zhu M-Y, Zhuravlev AY, Wood RA, Zhao F, Sukhov SS (2017). A deep root for the Cambrian explosion: Implications of new bio- and chemostratigraphy from the Siberian Platform. Geology.

[71] Kouchinsky A (2017). Terreneuvian stratigraphy and faunas from the Anabar Uplift, Siberia. Acta Pal. Pol..

[72] Telford MJ, Budd GE, Philippe H (2015). Phylogenomic insights into animal evolution. Curr. Biol..

[73] Zhao F-C, Smith MR, Yin Z-J, Zeng H, Zhu M-Y (2017). High resolution images of *Orthrozanclus elongata*. FigShare.

